# May-Thurner Syndrome: The Worst-Case Scenario

**DOI:** 10.7759/cureus.55742

**Published:** 2024-03-07

**Authors:** Marta Costa, Gonçalo Ferreira, Dora Gomes, Catarina Oliveira, Nelson Domingues

**Affiliations:** 1 Department of Internal Medicine, Unidade Local de Saúde Viseu Dão-Lafões, Viseu, PRT; 2 Department of Cardiology, Unidade Local de Saúde Viseu Dão-Lafões, Viseu, PRT; 3 Department of Internal Medicine, Unidade Local de Saúde Viseu Dão-Lafões, viseu, PRT

**Keywords:** heart arrest, pulmonary embolism, vascular malformations, cardiovascular abnormalities, may-thurner syndrome

## Abstract

May-Thurner syndrome (MTS) is caused by compression of the left common iliac vein by the right common iliac artery against the spinal column. It can range from asymptomatic or present with subtle and unspecific signs and symptoms and rarely exhibit severe complications such as pulmonary embolism (PE). The diagnosis is confirmed by typical imaging findings. Treatment may include conservative measures, anticoagulation, endovascular or even surgical options. We report the case of a 20-year-old female who presented with cardiac arrest caused by an acute massive PE. Further study showed partial thrombosis of the internal iliac veins resulting from MTS. She continued anticoagulation therapy with low-molecular-weight heparin and then switched to edoxaban with a good clinical outcome. She was also referred to Vascular Surgery to discuss the possibility of iliac vein stenting. Abdominopelvic vascular compression syndromes include a large spectrum of conditions, and they are rarely considered as an etiology for venous thromboembolism. The clinical presentation of PE varies with several triggering factors and atypical presentation is more common in nonmalignant causes. The combination of noninvasive and invasive imaging modalities might be beneficial to establish a definitive diagnosis. Nevertheless, invasive procedures are often restricted to doubtful cases or to guide endovascular procedures which is the current treatment of choice. There is little evidence using nonvitamin K oral anticoagulants, but there are some case reports detailing their successful use. This case aims to point out the need for a profound understanding of different causes of deep vein and pulmonary thromboembolism; common entities in our practice but with a variety of clinical presentations and potentially caused by rare underlying conditions. MTS can be the origin of serious and deadly complications, hence the importance of early recognition and treatment.

## Introduction

May-Thurner syndrome (MTS), also called Cockett syndrome or iliac vein compression syndrome, is caused by compression of the left common iliac vein by the right common iliac artery against the spinal column, more frequently the 5th lumbar vertebrae [1]. The exact incidence and prevalence are unknown since most patients are clinically asymptomatic [2], as venous collaterals can develop to maintain the blood flow or if the obstruction is not critical [3,4]. MTS has been implicated as the underlying etiology in 2% to 5% of lower extremities disorders [1] but several retrospective cadaveric and radiographic studies estimate a much higher prevalence [3]. It is known that MTS in children is rarely diagnosed when compared to adults [5] and that the incidence in women, usually between the 2nd and 4th decades of life [6], is two times higher than in men [3].

Any vessel can be compressed, but only when the vessels involved are linked to a corresponding image and clinical pattern we can call it a compression syndrome [7]. MTS develops throughout different stages as the chronic pressure and irritation of the endothelium by the artery’s pulsation leads to the formation of a venous spur that promotes clotting [3]. So far there are no studies showing a genetic predisposition to MTS [8]. It can range from asymptomatic or present with subtle and unspecific signs and symptoms like swelling, hyperpigmentation, telangiectasias, venous ulcerations, or deep vein thrombosis [6,9]. In severe cases, it can be associated with phlegmasia cerulea dolens or phlegmasia alba dolens. Complications of MTS include venous rupture, retroperitoneal hematoma and pulmonary embolism (PE) [10], and most frequently post-thrombotic syndrome (PTS) [3]. All of these possible clinical presentations only occur in the presence of transient risk factors such as surgery, pregnancy, post-partum [3], coagulopathy, obesity, long-term immobility, and estrogen-containing medication use such as oral contraceptive pills [5].

The diagnosis is confirmed by typical imaging findings on different methods such as ultrasound (US) Doppler or venography, through computerized tomography (CT), magnetic resonance imaging (MRI), or intravascular US (IVUS) [3]. Thrombophilia should also be excluded as literature reveals a correlation with MTS´s outcome [2,5].

Management of MTS involves a stepwise approach [2] and treatment will depend essentially on the degree of venous stasis and the presence of venous thrombosis [11]. Strategies may include conservative measures, anticoagulation, endovascular or even surgical options, mainly reserved for patients who fail endovascular procedures [3]. The latter may involve venous patch angioplasty, bypass with prosthetic or saphenous vein, lysis of adhesions, and creation of arteriovenous fistula [8].

## Case presentation

**Figure 1:**
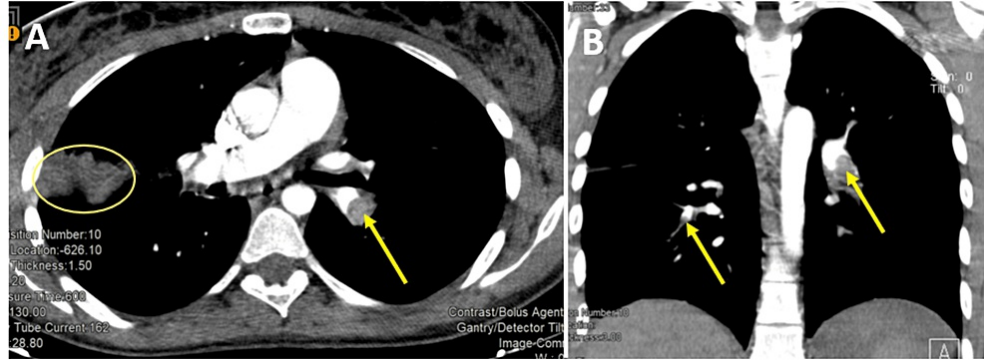
Bilateral Pulmonary Embolism in axial (A) and coronal (B) views Filling defect in the pulmonary arteries caused by the presence of thrombus (arrows) and an area of pulmonary infarction (circle).

We report the case of a 20-year-old female with a healthy and active life, no personal or family priors to report, medicated with combined oral contraceptive (ethinylestradiol 2 mg and chlormadinone acetate 0.03 mg per day), and no history of allergies. The patient presented with cardiac arrest and was promptly assisted with basic and advanced life support, with the return of spontaneous circulation after 14 minutes. When she first arrived at the emergency room, she was hemodynamically unstable needing aminergic and ventilatory support. A bedside transthoracic echocardiogram revealed right heart dilation suggesting PE, so she started fibrinolytic therapy with alteplase according to the hospital protocol. Later on, she performed a computed tomography pulmonary angiogram (CTPA) confirming an acute massive PE (Figures 1A, 1B).

She was admitted to the Intensive Care Unit (ICU) for further care with a favorable clinical evolution recovering from the initial organ failure and being able to deescalate care. Afterward, while in the general ward, the patient mentioned chest pain paroxysms with one-month evolution accompanied by exertional dyspnea and intense fatigue in the past week. There was no history of trauma or previous symptoms in the lower limbs suggestive of thrombus or phlebitis.

**Figure 2:**
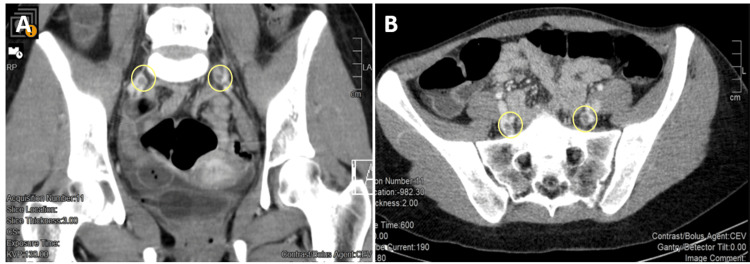
Thrombosis of the internal iliac veins in coronal (A) and axial (B) views Presence of hypodense material in the internal iliac veins suggesting thrombosis (circles).

The patient reported more episodes of chest pain during hospitalization with a good response to analgesic therapy. Additional investigation was conducted with a thorax, abdomen and pelvis CT scan that disclosed areas of pulmonary infarction and limited intra-alveolar hemorrhage after a vascular compromise, and also showed a partial thrombosis of the internal iliac veins (Figures 2A, 2B). Blood workup including coagulation and genetic study excluded thrombophilia or other obvious causes (Table 1). She also underwent echocardiographic reassessment that exhibited good systolic function and a low probability of pulmonary hypertension.

**Table 1:** Hipercoagulable workup * DRVVT - dilute Russell viper venom test

HIPERCOAGULABLE TESTS	RESULTS	REFERENCE VALUES
Leukocytes	7.91x10^9^	4.5-11.5 x 10^9^/L
Hemoglobin	11.8	12-15 g/dL
Platelets	295x10^9^	150-450 x 10^9^/L
Prothrombin time	16.2	11.7-15.3 seconds
Prothrombin activity (%)	72	70%-100%
INR (International Normalized Ratio)	1.2	0.8-1.1
Activated partial thromboplastin time (APTT)	32.2	25-34 seconds
D-dimer	5067	<500 ng/mL
Factor V Leiden	Negative	Negative
Prothrombin G20210A	Negative	Negative
Protein C	64	70%-140%
Protein S	47	54.7%-123.7%
Antithrombin III	87	83%-128%
Lupus anticoagulant (APTT and DRVVT*)	Negative	Negative
Anticardiolipin IgG	0.7	0-10 U/mL
Anticardiolipin IgM	< 0.9	0-10 U/mL
Beta2-glycoprotein I IgG	1.1	0-10 U/mL
Beta2-glycoprotein I IgM	5	0-10 U/mL

After the initial fibrinolysis, she continued anticoagulation therapy with low-molecular-weight heparin while hospitalized and switched to edoxaban, a direct oral anticoagulant (DOAC), by the time of her discharge after two weeks of hospitalization, with a good clinical outcome and no reported side effects.

**Figure 3:**
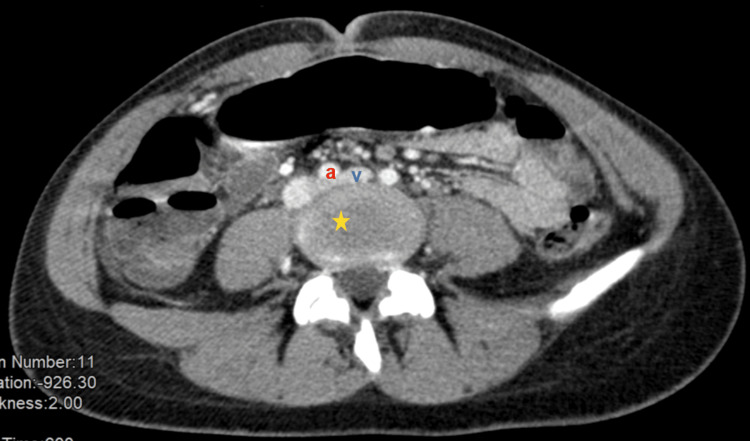
May-Thurner syndrome Compression of the left common iliac vein (v) by the right common iliac artery (a) against the lumbar vertebrae (*).

She was scheduled for a follow-up appointment where she complained of new onset paresthesia in her left lower limb. An additional abdomen and pelvis CT scan unveiled compression of the left common iliac vein caused by the right iliac artery indicating MTS (Figure 3). She was advised to maintain anticoagulation treatment and was without delay referred to Vascular Surgery to discuss the possibility of iliac vein stenting.

## Discussion

Abdominopelvic vascular compression syndromes include a large spectrum of conditions, and their exact prevalence is unknown [6]. They are rarely considered as an etiology for venous thromboembolism [12], and in many cases, the iliac vein compression is not properly diagnosed [10]. This recognition is vital not only to treat possible complications such as PE in MTS, where a prompt diagnosis is of utmost importance to patient survival [13] but also to prevent vascular injuries during other surgical procedures [6]. Additionally, clinical presentation of PE varies with several triggering factors; some studies show that atypical presentation is more common in nonmalignant causes [13]. In this particular case, we found that hypercoagulability was probably due to the oral contraceptives that the patient was taking therefore completing Virchow's triad to clot formation [4].

We already established that we have different imaging techniques at our display capable of detecting MTS, although it is prudent to remember that, despite the different advantages, all of them also present some limitations. US Doppler for example is the most common technique used to diagnose a DVT but presents technical difficulties in assessing the inferior vena cava (IVC) and iliac veins limiting its use in this case [3]. On the other hand, CT venography not only has a higher sensitivity and specificity to detect iliac vein compression, but it is also helpful in ruling out other causes. All of these gains and the possibility to perform different protocols, according to the specific syndrome to maximize the visibility, make CT the most recommended imaging method providing accurate detection of vascular structures and their relationship with adjacent organs. MRI can be an alternative especially in children or young patients due to the absence of ionizing radiation [6].

The combination of noninvasive methods, that allow the precise evaluation of anatomical structures, and invasive techniques, that are useful for the direct measurement of pressure gradients, is beneficial to establish a definitive diagnosis. Nonetheless, invasive procedures are often restricted to doubtful cases or to guide endovascular procedures [6]. IVUS remains the gold standard since it provides a real-time evaluation of the vessel lumen, the accurate luminal diameter, structural changes in the vessel wall, and information regarding the chronicity of the thrombus. In addition, to help in deciding management, the biggest benefit is that contrast is not needed in venous studies, decreasing the chances of contrast-related complications [3].

The therapeutic approach to MTS has evolved over the past few decades favoring endovascular management [12], with the current treatment of choice being venography IVUS-guided endovascular stenting [8]. There are no specific guidelines and the treatment is largely at the physician’s discretion so cases like this strongly benefit from a multidisciplinary discussion given their complexity and lack of solid recommendations. First-line therapy for patients with non-thrombotic MTS might be daily compression stocking use, exercise, and weight loss, while anticoagulation and consideration of endovascular therapy are standard for thrombotic MTS [8].

Patients are generally started on anticoagulation to prevent recurrence and post-stenting stent thrombosis. Underlying thrombotic and bleeding risk factors will posteriorly guide the extension of the treatment, even though the exact duration is not yet standardized. Even though there are only a few studies regarding this topic, warfarin seems to be the anticoagulant of choice in most papers and there is little evidence using nonvitamin K oral anticoagulants, but there are some case reports detailing the successful use of DOAC, mainly with rivaroxaban [9]. The goal of intervention is to reduce long-term sequelae, primarily the development of PTS. Clinical outcomes are variable and may depend on patient and anatomical factors, as well as symptom chronicity at presentation [8].

Nowadays, the acknowledgement of MTS has increased due to physician awareness leading to a reinforcement of literature and improved imaging capabilities. The role of endovascular therapy has expanded significantly and changed the paradigm of MTS management [8].

## Conclusions

The report of this unusual case aims to point out the need for a profound understanding of different causes of deep vein and pulmonary thromboembolism, common entities in our practice but with a variety of clinical presentations and potentially caused by rare underlying conditions. We also intended to emphasize the need for a high level of suspicion to pursue a targeted and full investigation leading to an exceptional diagnosis as MTS might be.

Although it may well be asymptomatic, MTS can also be the origin of serious and deadly complications as this case kindly illustrates, hence the importance of early recognition and treatment. The increasing research has been and hopefully will keep improving to provide virtuous care to our patients.
